# “Is there anything good about a water advisory?”: an exploration of the consequences of drinking water advisories in an indigenous community

**DOI:** 10.1186/s12889-020-09825-9

**Published:** 2020-11-13

**Authors:** Kayla J. Lucier, Corinne J. Schuster-Wallace, Derek Skead, Kathleen Skead, Sarah E. Dickson-Anderson

**Affiliations:** 1 Department of Health Research Methods, Evidence, and Impact, McMaster University, Hamilton, Ontario L8S 4L8 Canada; 2 Department of Geography and Planning / Global Institute for Water Security 12 Kirk Hall Building, 117 Science Place, University of Saskatchewan, Saskatoon, Saskatchewan S7N 5C8 Canada; 3 Department of Civil Engineering, McMaster University, Hamilton, Ontario L8S 4L7 Canada; 4 Wauzhushk Onigum First Nation, Kenora, Ontario P9N 3X8 Canada

**Keywords:** Drinking water, Drinking water advisories, Indigenous health, First Nations, Community-based participatory research, Traditional knowledge, Canada

## Abstract

**Background:**

In Ontario, Canada, Indigenous communities experience some of the province’s worst drinking water, with issues ranging from deteriorating water quality to regulatory problems and lack of support. When water is known, or suspected, to be unsafe for human consumption, communities are placed under a Drinking Water Advisory. Between 2004 and 2013, approximately 70% of all on-reserve communities in Ontario were under at least one Drinking Water Advisory. Despite the widespread impact of Drinking Water Advisories on health and wellbeing, little is known about First Nation individuals’ perceptions and experiences living with a Drinking Water Advisory. This study presents information shared by members of a community who have lived with Boil Water Advisories on and off for many years, and a long-term Boil Water Advisory since 2017. The goal of this paper is to unpack and explore the Boil Water Advisories from the perspective of community members and provide considerations for current and future Boil Water Advisory management.

**Methods:**

Methodological choices were driven by the principles of community-based participatory research. Two data collection methodologies were employed: hard copy surveys and interviews.

**Results:**

Forty-four individuals (19.5%) completed a survey. Eight Elders and 16 key informants participated in 20 interviews. Respondents expressed varying degrees of uncertainty regarding protective actions to take while under a Boil Water Advisory. Further, 79% of men but only 46% of women indicated they always adhere to the Boil Water Advisory. Knowledge gaps that could lead to risky behaviours were also identified. Finally, Boil Water Advisories were demonstrated to have physical, financial, and time impacts on the majority of respondents.

**Conclusions:**

A direct outcome was the identification of a critical need to reinforce best practices for health protection through community education and outreach. More broadly, Chief and Council were able to use the findings to successfully advocate for improved drinking water for the community. Additionally, benefits of participatory research and community ownership include enhanced local research capacity, and increased awareness of, and desire for, research to inform decisions.

**Supplementary Information:**

The online version contains supplementary material available at 10.1186/s12889-020-09825-9.

## Background

In Ontario, Canada, Indigenous (on-reserve) communities face drinking water insecurity [1]. Issues range from deteriorating source water quality to ineffective, inappropriate, and aging infrastructure, regulatory challenges, and a lack of support for local water management and treatment operators [1, 2]. Between 2009 and 2011, 73% of First Nations water systems were considered medium- to high-risk for producing unsafe drinking water [3].

When water is known, suspected to be, or could become, unsafe for human consumption, communities are placed under a Drinking Water Advisory (DWA). The type of DWA depends on the nature of the problem, with advisories ranging from boiling tap water before consuming (Boil Water Advisory, BWA) to not consuming or using tap water under any circumstances (Do Not Consume Advisory). DWAs can be issued for any community in Canada but are more complicated in the Indigenous context in Ontario. This is because water systems in Ontario must meet provincial standards rather than federal guidelines. However, despite being located in Ontario, reserves are under federal jurisdiction because they are on Crown Land. Thus, the onus for water treatment and management falls on Indigenous communities and the Government of Canada, specifically Indigenous Services Canada (ISC) and Health Canada (HC) [4]. ISC support is primarily financial or technical, while HC advises and supports Chief and Council in making decisions about water quality and monitoring. This decision-making is based on the Guidelines for Canadian Drinking Water Quality [4], not the more stringent Ontario Safe Drinking Water Act (2002), although communities may opt to apply the latter. Additionally, since 2016, technical support for reserves in Ontario is provided through Ontario’s Indigenous Drinking Water Projects Office (IDWPO), established through a tri-lateral technical working group. Ultimately, when a concern arises, it is Chief and Council who are responsible for issuing a DWA in their community based on the information available and for rescinding it after the problem has been rectified. Although the Federal Government does have a Protocol for Safe Drinking Water for First Nations Communities and a Safe Drinking Water for First Nations Act, it has been noted that the complicated government structure involving ISC, HC, and IDWPO leads to insufficient drinking water regulation on reserves [5].

The Indigenous framing of human health and wellness differs from the Western biomedical model, which sees health as the absence of disease [6]. In many Indigenous models of health, there are four components to wellbeing – physical, mental, emotional, and spiritual health [7]. The terms “holism” or “holistic” are often used when describing the four components of Indigenous health because each of the four elements must be balanced or aligned and “free from complications, limitations, and frustrations” [7] for a person to be in optimal health. A connection to nature and the land is also an important component of health; this has been explained as “if the land is well the people will be well” [7].

Many Indigenous peoples have important and long-lasting relationships with water; issues related to water quality and availability impact these relationships, thus impacting an essential facet of Indigenous life [8]. Many Indigenous stories also support that water is first medicine and has healing properties [9]. As such, water is not just life-sustaining – water *is* life and water *has* life or a spirit [5, 10] and, as a living entity, it has the ability to form relationships and engage with others [8]. Although women have a special connection to water, there is a shared responsibility among all people to care for Mother Earth, including, and especially, her waters [10, 11].

Between 2004 and 2013, 402 DWAs were issued in First Nation communities in Ontario alone, with approximately 70% of all First Nations under at least one DWA [12]. In 2011, First Nations in Canada were reportedly 2.5 times more likely to be under a BWA than non-Indigenous communities [13]. As of February 2020, 61 long-term (i.e. 1 year or longer) and 25 short-term DWAs in ISC-funded reserves in Canada were still standing [14, 15]. An additional 11 DWAs in non-public, non-ISC funded drinking water systems on reserves were also in place [16]. Despite DWAs impacting many people living on-reserve in Ontario, little is known about First Nation residents’ experiences living under a BWA. The goal of this paper is to unpack and explore the BWA from the perspective of community members’ lived experiences and self-reported impacts in order to provide considerations for current and future BWA management.

## Methods

This study was undertaken in Wauzhushk Onigum First Nation, an Anishinaabe First Nations community on the shores of the Lake of the Woods, Ontario, Canada. The community is currently under a BWA as a result of a drinking water treatment plant that does not meet provincial standards. Where they live holds significant cultural and traditional value for the community, even though ambient water quality has been steadily declining. This community faces many infrastructure, source water protection, and water management challenges and has lacked the resources to deal with these barriers.

The study design used a community-based participatory research (CBPR) approach. CBPR is not a prescriptive research methodology, but an approach to conducting research that influences all aspects of the research process and from which methodologies flow [17]. CBPR is a preferred approach to undertaking research with Indigenous people because of the community participation, education, action, and capacity-building included within the pursuit and production of knowledge [18, 19]. This is in contrast to a long history of research by and with Indigenous people and communities that has been, and oftentimes continues to be, guided by a non-Indigenous, Euro-Christian, and colonial worldview [20, 21] and years of systemic social, political, and economic oppression in Canada [22]. As such, the adoption of CBPR with Indigenous communities contrasts a history of marginalization through engagement in collaborative research that builds research capacity and privileges Indigenous voices. Although CBPR is not exclusively Indigenous, its approaches are consistent with Indigenous research paradigms [19] and it responds to the recommendations of the Truth and Reconciliation Commission of Canada [22]. Combining CBPR with the 4 R’s, or core values, of Indigenous research (i.e., respect, responsibility, reciprocity, and relevance) [23], ensure that research is conducted in a meaningful way with communities and that the interests of Indigenous populations are not lost within the research process.

The community and academic partners were introduced by a third party, who perceived that collaborative opportunities existed. After several informal discussions that served to build trust and relationships over a period of several years, the researchers were invited to discuss specific research opportunities related to community water challenges with Chief and Council. The research question was determined through these discussions with Chief and Council. Data collection tools were collaboratively designed by co-authors and revised through an iterative process with Chief and Council. Data analyses also followed this iterative process with Chief and Council and community Elders. As such, community leadership determined research questions, methodologies, and outcomes with the research team as collaborators, rather than the other way around. Data collection was undertaken by a graduate student and community research associate (CRA). The graduate student resided in the community for the duration of data collection, and all academic researchers attended community events, feasts, ceremonies, and celebrations outside the scope of the research. This was important to community members and helped build mutual respect. The engagement of the CRA, with lived experience in the community, in the research was critical to ensuring ongoing research relevance and community support as well as increasing the long-term community capacity to engage in research and the empowerment of self [24]. Prior to engagement with the community, community Elders conducted a water ceremony. The data collection period was preceded by a community feast, during which the project genesis, process, and participation were explained and members given the opportunity to ask questions.

When engaging in CBPR, it is important to recognize that who we are and where we come from drives our interests, fields of study, and collaborative approaches and therefore how we collect and interpret data [25]. Authors represent Indigenous Anishinaabe, with a broad understanding of Indigenous Traditional Knowledge (TK) and experience living under a BWA (DS, KS) and non-Indigenous, allied, settler scholars with different disciplinary backgrounds in water-related health (KJL, CJSW, SEDA). As such, multiple perspectives were brought to bear on all aspects of the research process.

This research received ethics approval from the McMaster Research Ethics Board (Certificate #2017 197) and the University of Saskatchewan Research Ethics Board (Certificate #504). Two data collection methods were employed: surveys and semi-structured interviews. Each person 18 or older living in the community was eligible to participate in the survey, which consisted of sections that focused on: 1. Basic information about the respondent (e.g., age; education); 2. Knowledge of the BWA (e.g., How did the respondent hear about the BWA? Did they seek more information about the BWA?); 3. Water sources and uses before and during the BWA (e.g., What was/is the source of respondent’s drinking water? What was/is the source of water for food preparation?); and, 4. Impacts of the BWA upon the respondent’s life (e.g., What was the financial impact of the BWA on respondent and their family? What was their emotional response to the BWA?). The survey instrument is provided in an additional file (Additional file 1.pdf). To ensure that each person was given only one survey, a log was kept with the name of each recipient and, if possible, their street and house number. The study team was available to assist with completing the surveys through interviews for anyone who requested, recording verbal responses on the physical surveys. An Ojibwe translator was also made available. Surveys were returned in envelopes to protect anonymity, particularly if community members decided to return a blank survey.

At the community’s request, return of completed surveys was incentivized through a raffle for a tablet computer. The winning participant was drawn by a non-community member working out of the Band Office and was discreetly contacted and given their prize to maintain participant anonymity. Surveys were coded and analyzed using Excel©.

Semi-structured interviews were used to solicit important contextual information from key informants (KIs) internal and external to the community. The interview instrument is provided in an additional file (Additional file 2.pdf). KIs held roles related to water quality testing, management, and operations, Band management and operations, and community health and wellness. These individuals were identified by community partners and Chief and Council. Elders were also interviewed and given tobacco and honoraria in recognition of their TK according to local cultural practices. Sixteen KIs and eight Elders participated in a total of 20 interviews, as some KI interviews had more than one person participating. All but one interview was conducted in-person. Where permitted, interviews were recorded, with recordings transcribed verbatim and anonymized. Interview transcripts were imported into NVivo 12© for analysis. Interviews were initially coded according to questions asked in interviews and emerging themes. An inductive approach was taken to develop the final code set once the initial coding hierarchy had been created. KI and Elder interviews were coded separately as each offered unique perspectives.

Academic research team members synthesized preliminary findings between November 2018 and May 2019. These preliminary findings were co-analysed in June 2019 after a second water ceremony, and included separate meetings with Chief and Council, Elders, and the community at large to review the findings of the research they requested. Prompts to guide discussions included: “Does this make sense?”, “What jumps out and why?” and “Why do you think community members said what they said?”. As part of the analysis, data were disaggregated by gender. Data were not disaggregated by survey age categories because some were very small and not reflective, proportionally, of that age group. However, data were disaggregated by younger (18–49) and older (50 and above). The final results were reported back to the community at another feast.

## Results

Two hundred and twenty-six surveys were distributed to eligible participants throughout the community; 44 (19.5%) were returned completed and 11 (4.9%) were returned blank. Fifteen Elders and 22 KIs were contacted through written and verbal invitation. The data are presented according to the five major themes of the survey: 1) Community Context 2) Knowledge of BWAs; 3) Living Under a BWA; 4) Water and Health; and, 5) Pathways Forward.

Survey respondents were split fairly evenly in terms of gender, with 43% (*n* = 19) identifying as men and 55% (*n* = 24) identifying as women (Table 1). The majority of respondents (*n* = 33, 75%) were over the age of 40 and had lived on the reserve for at least 10 years (*n* = 37, 84%). Just over half of respondents (*n* = 24, 55%) had lived elsewhere at some point in time, mainly for work or studies. Almost two-thirds of respondents (*n* = 25, 57%) indicated that their highest level of formal education was high school/GED and one-fifth had completed post-secondary education (*n* = 9, 21%). Respondents also commented on many traditional forms of education, including family knowledge (*n* = 4, 9%), Elders (*n* = 2, 5%), medicine teachings (*n* = 2, 5%), and living off the land (*n* = 2, 5%).

**Table 1:** Survey Respondent Demographic Information

	n (%)
**Gender (*N*** **= 44)**	
Men	19 (43)
Women	24 (55)
**Age (*N*** **= 44)**	
18–29	4 (9)
30–39	6 (14)
40–49	14 (32)
50+	19 (43)
**Years Living in Community (*N*** **= 44)**	
< 1 year	0 (0)
1 to < 3 years	1 (2)
3 to < 5 years	2 (5)
5 to < 10 years	2 (5)
≥ 10 years	37 (84)
**Lived Elsewhere? (*N*** **= 44)**	
Yes	24 (55)
No	18 (41)
**Highest Formal Education (*N*** **= 44)**	
Elementary School	0 (0)
High School/GED	25 (57)
Vocational Training	3 (7)
University/College	9 (21)
Graduate Degree/Professional Credentials	2 (5)

Elders and KIs were asked to reflect on causes of BWAs in the community. Reasons identified included: companies contracted by the government to put water plants on reserves without ensuring that they are appropriate, or without allocating “money to keep [them] running properly” (KI15); a lack of “regulatory infrastructure” on reserve (KI15); the use of reserve land to haul oil or other harmful substances (E10); and, larger populations (e.g., in towns) having more expensive water treatment plants (KI5).

Not everyone demonstrated the same level of awareness of the current BWA (Table 2), despite the identification of several avenues of BWA notification such as the Band Office (*n* = 8, 18%) and community fliers, posters, or bulletins (*n* = 7, 16%). A significant number of respondents indicated that they always consider themselves under a BWA (*n* = 6, 14%) because they are never sure if there is one in place or not - “[I] never drink uh, water from the tap because [I] don’t know whether it’s uh, it’s on or not” (E9). It should be noted that a lack of access to the Internet was perceived to be a barrier to receiving BWA notifications.

**Table 2:** Knowledge of Practices When Under a BWA by Gender^a^

	Yes	No	I Don’t Know	Blank
	n (%)	n (%)	n (%)	n (%)
*Applies only to my tap water*				
Men (*N* = 19)	14 (74)	5 (26)	0 (0)	0 (0)
Women (*N* = 24)	14 (58)	1 (4)	5 (21)	4 (17)
*I can drink my tap water as long as it is clear*				
Men	3 (16)	16 (84)	0 (0)	0 (0)
Women	4 (17)	16 (67)	4 (7)	0 (0)
*I should not drink my tap water*				
Men	15 (79)	3 (16)	0 (0)	0 (0)
Women	14 (58)	8 (33)	1 (4)	1 (4)
*I should boil my tap water to prepare food*				
Men	15 (79)	3 (16)	0 (0)	0 (0)
Women	17 (71)	5 (21)	2 (8)	0 (0)
*I can use my tap water to make ice*				
Men	3 (16)	16 (84)	0 (0)	0 (0)
Women	4 (17)	19 (79)	1 (4)	0 (0)
*I can bathe/shower using my tap water*				
Men	18 (95)	0 (0)	1 (5)	0 (0)
Women	23 (96)	1 (4)	0 (0)	0 (0)
*I can give my babies and young children a sponge bath using my tap water*				
Men	10 (53)	4 (21)	2 (11)	3 (16)
Women	15 (63)	5 (21)	2 (8)	1 (4)
*Before preparing meals, it is okay to wash my hands with tap water*				
Men	14 (74)	5 (26)	0 (0)	0 (0)
Women	20 (83)	2 (8)	2 (8)	0 (0)
*I can use my tap water to clean my teeth*				
Men	12 (63)	5 (26)	1 (5)	1 (5)
Women	18 (75)	3 (13)	3 (13)	0 (0)
*I should boil my tap water before I drink it*				
Men	15 (79)	3 (16)	0 (0)	1 (5)
Women	18 (75)	5 (21)	1 (4)	0 (0)
*I should boil my tap water before I bath my babies and young children*				
Men	7 (37)	8 (42)	3 (16)	1 (5)
Women	4 (17)	8 (33)	9 (38)	2 (8)
*I can use a Brita filter to decontaminate my tap water*				
Men	10 (53)	6 (32)	2 (11)	1 (5)
Women	7 (29)	8 (33)	8 (33)	1 (4)
*I should drink bottled water*				
Men	19 (100)	0 (0)	0 (0)	0 (0)
Women	23 (96)	1 (4)	0 (0)	0 (0)

^a^ excluding those who preferred not to disclose gender

Respondents expressed varying degrees of uncertainty regarding what protective actions should and should not be undertaken while under a BWA. The most uncertainty was around whether tap water should be boiled before bathing babies and young children (*n* = 12, 27% did not know), or if a Brita filter is effective in decontaminating tap water (*n* = 10, 23% did not know). Some respondents identified knowledge gaps that could lead to risky behaviours, believing that they could continue to drink their tap water as long as it is clear (*n* = 7, 16%), use their tap water to make ice (*n* = 7, 16%), or use their tap water to clean their teeth (*n* = 31, 70%), all of which are potential transmission routes for disease, and advised against while under a BWA. An additional 39% (*n* = 17) believed that a Brita filter would purify their tap water, when in fact it does not remove microbiological contaminants. When looking at knowledge of practices by gender, men were more likely to correctly identify recommended practices than women. For example, 84% (*n* = 16) of men but only 67% (*n* = 16) of women correctly identified that they could not drink their tap water even if it was clear, and, proportionally, twice as many men than women correctly identified they could not use their tap water to brush their teeth (Table 2).

When asked about their water sources and uses (Table 3), responses of bottled and tap water changed by approximately 20% when under a BWA versus not, for drinking, food preparation, and cooking. While under a BWA, over half of responses (*n* = 28, 58%) indicated that respondents use tap water for cleaning their teeth, aligning with the knowledge gap identified previously, and demonstrating that lack of knowledge leads to risky behaviours. Approximately one-quarter of responses indicated that participants use tap water for food preparation and cooking (*n* = 11, 23% and *n* = 13, 27%, respectively), with 6% (*n* = 3) using lake water, and one respondent using boiled water. Three KIs identified that they could boil their tap water to make food, soup, and coffee, which makes it difficult to ascertain whether survey respondents were referring to using tap water for all food preparation and cooking, which could pose a health risk, or just for foods that could be boiled.

**Table 3:** Water Sources and Uses

Where do you get your water for the following purposes?	Tap %	Lake %	Bottled %	Well %	Spring %	Boiled %	Blank %
**Drinking**							
No BWA (*N* = 52)	27	6	63	2	0	0	0
BWA (*N* = 46)	9	0	87	2	0	0	2
**Food Preparation**							
No BWA (*N* = 47)	43	9	45	2	0	0	2
BWA (*N* = 48)	23	6	65	2	0	2	2
**Cooking**							
No BWA (*N* = 49)	45	8	43	2	0	0	0
BWA (*N* = 48)	27	6	60	2	0	2	2
**Cleaning Teeth**							
No BWA (*N* = 49)	61	14	22	2	0	0	0
BWA (*N* = 48)	58	8	29	2	0	0	2
**Hand Washing**							
No BWA (*N* = 50)	70	22	6	2	0	0	0
BWA (*N* = 47)	70	15	11	2	0	0	2
**Bathing**							
No BWA (*N* = 50)	74	22	2	2	0	0	0
BWA (*N* = 47)	75	15	6	2	0	0	2
**Cleaning**							
No BWA (*N* = 50)	76	22	0	2	0	0	0
BWA (*N* = 46)	76	13	7	2	0	0	2
**Laundry**							
No BWA *(N* = 49)	78	20	0	2	0	0	0
BWA (*N* = 46)	80	13	0	2	0	0	2
**Ceremonies**							
No BWA (*N* = 47)	32	6	26	2	2	0	11
BWA (*N* = 46)	17	7	33	2	2	0	15

The majority of respondents (*n* = 27, 61%) indicated that they always adhere to the BWA, while 36% (*n* = 16) occasionally adhere (i.e., those who sometimes adhere, adhere when convenient, or rarely adhere) and one indicated that they never adhere. When stratified by gender, there was a clear difference in reported adherence between men and women. While 79% (*n* = 15) of men indicated they always adhere to the BWA, less than half of women (*n* = 11, 46%) said the same. However, age also played a role in reported adherence (Table 4). A greater percentage of older women reported that they always adhere compared to younger women.

**Table 4:** Self-Reported Adherence to a DWA by Gender and Age (younger is 18–49; older is 50 and above)^a^

Adherence to BWA (*N*= 44)	Always Adhere n (%)	Occasionally Adhere n (%)	Never Adhere n (%)
Younger Men (*N* = 11)	8 (73)	3 (27)	0 (0)
Younger Women (*N* = 12)	4 (33)	7 (58)	1 (8)
Older Men (*N* = 7)	6 (86)	1 (14)	0 (0)
Older Women (*N* = 12)	7 (58)	5 (42)	0 (0)

^a^ excluding those who preferred not to disclose age or gender

Clear disconnects emerged between knowledge and practice. For example, 4% (n = 2) of respondents indicated that they always adhere to the BWA, while also indicating that they use tap water for drinking during a BWA. Other respondents indicated that they always adhere to the BWA, but use tap water for food preparation (*n* = 5, 11%) or cooking (*n* = 6, 13%) under a BWA although, as previously indicated, some ambiguity may exist around whether this extended to all food types. The largest disconnect between knowledge and practice was around teeth cleaning; 39% (*n* = 18) of respondents indicated that they always adhere to the BWA, but also indicated that they used tap water for cleaning their teeth. Some people clearly expose themselves to risk unnecessarily while believing that they are adhering to the BWA. A risky practice that has not caused illness in the past is still a pathway for disease in the future.

Respondents were asked to describe impacts on health (physical, emotional, psychological, spiritual), finances, and time as a result of living under a BWA. Eighty-nine percent (*n* = 39) of respondents indicated at least some impact and 59% (*n* = 26) reported significant or high impact in one or more categories (Table 5). One KI further commented on domestic tensions related to whose turn it was to collect the bottled water (KI20). Several spoke of problems with an insufficient supply of bottled water (KI7, KI11, KI16, KI19) and individuals “taking more than they’re … allotted” (KI20) so that others did not have enough water on a weekly basis (E9, KI11, KI19).

**Table 5:** Impacts of BWA by Gender or Age

Negtaive Impacts of BWA	Significant/High Impact	Some Impact	Little/No Impact	Blank
	n (%)	n (%)	n (%)	n (%)
**Financial**				
Men (*N* = 19)	6 (32)	9 (47)	3 (16)	1 (5)
Women (*N* = 24)	3 (13)	10 (42)	8 (33)	3 (13)
Younger (*N* = 24)	5 (21)	12 (50)	6 (25)	1 (4)
Older (*N* = 19)	4 (21)	7 (37)	5 (26)	3 (16)
**Physical**				
Men	6 (32)	9 (47)	4 (21)	0 (0)
Women	10 (42)	5 (21)	7 (29)	1 (4)
Younger	11 (46)	8 (33)	4 (17)	0 (0)
Older	6 (32)	5 (26)	7 (37)	1 (5)
**Psychological**				
Men	5 (26)	6 (32)	7 (37)	1 (5)
Women	7 (29)	4 (17)	10 (42)	3 (13)
Younger	9 (38)	4 (17)	10 (42)	1 (4)
Older	4 (21)	5 (26)	7 (37)	3 (16)
**Social**				
Men	5 (26)	3 (16)	11 (58)	0 (0)
Women	7 (29)	3 (13)	11 (46)	3 (13)
Younger	10 (42)	2 (8)	11 (46)	1 (4)
Older	3 (16)	3 (16)	11 (58)	2 (11)
**Spiritual**				
Men	9 (47)	2 (11)	7 (37)	1 (5)
Women	4 (17)	5 (21)	11 (46)	4 (17)
Younger	9 (38)	4 (17)	10 (42)	1 (4)
Older	5 (26)	2 (11)	8 (42)	4 (21)
**Time Burden**				
Men	6 (32)	7 (37)	5 (26)	1 (5)
Women	11 (46)	6 (25)	5 (21)	1 (4)
Younger	11 (46)	7 (29)	4 (17)	0 (0)
Older	7 (37)	5 (26)	6 (32)	1 (5)

Only 9% (*n* = 4) of respondents did not report any adverse impacts. It is important to note that responses differed according to gender and age (Table 5). Proportionally, more women than men and more younger than older respondents indicated significant or high physical (gender: *n* = 10, 42% vs. *n* = 6, 32%; age: *n* = 11, 46% vs. *n* = 6, 32%) and time (gender: *n* = 11, 46% vs. *n* = 6, 32%; age: *n* = 11, 46% vs. *n* = 7, 37%) impacts. The greatest disparity across age categories was with respect to social impacts, where 42% (*n* = 10) of younger respondents indicated significant or high impacts, while only 16% (*n* = 3) of older respondents said the same. More men than women reported significant or high spiritual impacts (*n* = 9, 47%; *n* = 4, 17%, respectively). Finally, women were more likely to report little or no impact financially compared to men (*n* = 8, 33%; *n* = 3, 16%, respectively), but impacts did not differ across younger and older respondents.

Many stories were shared about experiences with waterborne diseases, especially those causing diarrhoea. While one KI indicated that there were no recent occurrences of reportable waterborne diseases (KI18), another reported hearing of people contracting giardiasis and cryptosporidiosis and needing antibiotics (KI11). *Helicobacter pylori*, a pathogen associated with poor drinking water quality and sanitation that can lead to chronic stomach and digestive issues [26], was referenced by survey respondents (*n* = 3, 7%) and KIs (*n* = 2). Other health concerns identified as resulting from community water problems included hair loss (E8) and itchy skin (E1) or eczema (*n* = 2, 5%). One KI linked health effects to “whatever they put in the in the water” (K12). Four Elders and KIs extended water-related health to wildlife, referring to “fish with lumps on bodies” (E8) and fish “full of mercury” (E9). As expanded in the discussion section, this speaks to the community’s worldview of interconnectivity. When water quality impacts animals it also impacts their human counterparts: “They drink the water, they get sick and then we eat the meat, we get sick” (KI12). In this context, the relationship goes beyond physical health and encompasses environmental stewardship:

“You respect all life right from the tiniest insect to the biggest animal and they’re here too for a reason. And we all depend on water to survive. We all drink water. And that’s what we have to protect … all creation comes from water. And without the without the water we all die. Without the bugs we all die. Everything has a balance.” (KI12)

Most respondents described their emotional responses as worried (*n* = 26, 23%), unhappy (*n* = 20, 18%), and angry (*n* = 16, 14%). Other negative emotions identified included fear, stress, and confusion. Safety and happiness, which were also identified, may be seen as counterintuitive. However, the declaration of a BWA was seen by some to have inadvertent benefits. These included government awareness and facilitation, clean bottled water to drink, health protection, communication, and strengthened relationships:

“I think the project as a whole has actually brought everybody together uh, more closely because uhm, there’s a lot of, there’s a lot of action … I think it’s just sort of brought everybody together to, to work on the solution, so it’s, it’s been a positive experience.” (KI17)

While some relationships were strengthened, others were not. Not everyone in the community is serviced by the water treatment plant, which means that any solutions to the BWA will not improve drinking water for everyone. However, comments regarding disparities were not limited to within the community. Community members also looked outside their community to the local town and spoke to the drinking water inequities between them (E10, KI5, KI15).

Several solutions to the ongoing water issues were identified by survey respondents, Elders, and KIs. This is unsurprising, as it indicates a variety of valid avenues through which remediation can occur and through which individuals want to see action taken.

One solution was to resolve the BWA in traditional ways, such as turning to Mother Earth (E6), Elders (E8), or spirits:

“we talk to the to the entities, the spirits, entities whatever you want to call them, and they uh they set things right for us. And uh it’s something that science can’t explain. Sometimes I can’t explain it. But it happens.” (KI12)

Installation of point-of-use water filtration systems was another solution identified, particularly for homes that could not be connected to the community water distribution system (KI17). Other, less-cited, solutions included an unidentified “filter” (KI12) and finding a water source other than the lake (E6).

Cleaning the lake or stopping pollution was another solution (E2, E4, KI12) that included upgrading septic systems on the reserve (KI5). A role was identified for government in terms of involvement in finding, funding, and implementing a solution, partly because the reserve did not contaminate the water in the first place (E4). However, others saw this as requiring not only money, but also trust between the community and the government (KI5) and the nearby town (KI18), as well as a continued commitment to address BWAs on reserves (KI17, KI19, KI20). The solution articulated most frequently was to pipe water to the community from the closest town:

“Hooking up with the city of {name} is like bang, they’re there. They’re trained, they’ve got their qualifications they don’t go into a water plant unless they are qualified” (KI3)

It should be noted that this reflects an ongoing planning process that started after this study began. Many KIs noted that this solution was faster and less expensive to implement than other options such as upgrading the existing water treatment plant to meet provincial standards (*n* = 9). Though it would require less time and financial resources, piped water from the city was not always seen as the preferred solution over a community water treatment plant. One survey respondent illustrated this in a free-text comment:

“I hope that the water is drinkable someday … With the agreement to be connected to the {name} water system, I hope the work starts as soon as possible. I do however, think that we should have gotten our own water plant instead of connecting to {name}.” (S007)

Benefits of the pipeline were seen to include business and economic development opportunities (KI7, KI19, KI20). However, risks were also identified - one KI was scared that their water would be shut off if they did not pay their bill (KI5). At a higher level, this partnership was seen to create a reliance on the town as well as an undermining of autonomy. Other potential risks were identified, but seen to be mitigated already. For example, ISC was expected to bear the costs (KI5, KI20), and the current water treatment operators were expected to retain their employment (KI19).

Regardless of the final solution, there is an overwhelming desire:

“at the end of the day [to] have clean, safe drinking water for our membership so they could actually have that luxury of going to their tap, opening it up, and having a drink of water.” (KI19)

Research findings were reported back to the community in the form of an oral presentation and discussion, a written report, and a photo report, as requested. The community used the written report during negotiations with the closest town and ISC, which ultimately resulted in an agreement between the town and the community regarding the terms and conditions to connect the community to the municipal water treatment plant, and a commitment from ISC to cover the full cost of water usage by the First Nation, in addition to the original capital and maintenance cost of the infrastructure. Well drilling, for those who were unable to be connected to the piped water, will be started ahead of schedule. Originally the timeline for these connections was uncertain. Members of the community have postulated that this was ushered along faster than anticipated because of the unfairness between those who have and have not that emerged as a result of this research. In addition, the report helped the community health department identify the need for more bottled water for distribution and influenced the structure of bottled water delivery.

## Discussion

Census data from 2016 indicated that 300 people 15 years of age or older lived on the reserve. Of those, 80 (27%) were between 15 and 24 years old and 155 (52%) were 35 years of age or older [27]. This indicates that, although every effort was taken to provide all adults with the opportunity to participate, the average age of the sample was older than the average age of the eligible population. It was noted throughout the project that older community members in general appeared to share a greater interest in the study. It is possible that older individuals felt better situated to comment on the BWA considering the changes that they had witnessed over their lifetime. It is further possible that younger individuals purposefully chose not to participate out of respect for their Elders, who are considered to be the holders of TK, values, and teachings. The low response rate is likely the result of a decision to provide a single household perspective, which was articulated by at least one non-respondent. The surveys were distributed to approximately 110 households in the community. This indicates that household, rather than individual, surveys may be more appropriate in the future.

Given that, in general, women are more aware of the health implications of poor water quality, the differences in reported adherence to a BWA between men and women appear, on the surface, to be counterintuitive. There are several possible explanations for this finding. For example, a body of literature indicates that women have more ethical intentions than men, meaning they would be less likely to lie in their survey responses (reporting bias), although this is debated [28]. It may also be the result of differing roles and responsibilities between women and men [11, 29]. Women are responsible for child rearing, cooking, cleaning and maintaining the home – all tasks that are heavily water-dependent. Living under a BWA is a burden, and women may not have the luxury of adhering due to time and other constraints imposed by this burden. This is supported by the fact that older women were more likely to always adhere than younger women, who presumably carry the greater caretaking burden and were more likely to occasionally adhere (Table 4). In addition, proportionally more women than men and younger respondents than older respondents (Table 5) indicated significant or high negative physical and time impacts associated with the BWA.

Disconnects between reported adherence and practices may indicate a lack of understanding about the best practices to protect health under a BWA. Based on this lack of understanding, educational programs about practices that pose a risk to human health, and what one should do instead, are encouraged. Though this has the potential to benefit everyone, education aimed at women may have a larger impact given the gendered differences identified in this study.

With respect to differences in BWA impacts between younger and older respondents, younger people spend more time interacting socially [30]. If the BWA is taking up considerable time and energy that would normally be spent socializing, this could explain the differences in impact. Spirituality can take the form of learning more about one’s culture and values, practicing traditions, and using traditional medicines, practices which may be lost by the restrictions in place when living under a BWA [7, 31]. Indigenous women have a unique relationship with water. They are seen as the keepers and carriers of water for many reasons, including the connection between water and childbirth [32]. The Earth is also known as the “great Mother”, with her waterways carrying water similar to veins and arteries in people and animals [11]. Within this context, it is not surprising that men and women are impacted in different ways.

While self-reporting of illness is not always reliable [33], under-reporting of infectious gastrointestinal illnesses in Ontario is high, with each case reported to the province representing up to several hundred unreported cases depending on whether the illness is caused by a protozoa, bacteria, or virus [34]. Furthermore, many Indigenous communities, including this one, view health as more than the state of the physical body. Rather, health encompasses physical, spiritual, emotional, and psychological health [7]. Worry and anger are both negative emotions, but anxiety most often comes from a place of uncertainty and fear about handling an injustice, while anger comes from the need to blame someone for an injustice [35]. Collectively, the negative emotional responses points to the broader health implications of living under a BWA. Emotional wellbeing is an important component of Indigenous health; failure to address these negative emotions perpetuates poor health outcomes [7, 31]. Indigenous health is particularly linked to water, as from a spiritual perspective water has been referred to as “first medicine” [36].

The consideration for animal life points towards the equal importance of all living things [37], the strong reliance on country food [38] and the interconnectivity of land, water, people, and animals in Indigenous traditions related to wellness [37]. This community relies on a large surface water body for their drinking water source, and their conventional water treatment plant is primarily designed for pathogen removal, not heavy metals or persistent organic pollutants. While BWAs only focus on microbiological contamination, this may indicate that Do Not Consume (chemical, metal, or radionuclide contamination) management in Indigenous populations needs to include risk mitigation for wildlife and their consumption, because protecting animal wellbeing is a form of health promotion for humans.

The differences between those who have and those who have not is starker when it comes to a basic necessity such as water, even within a community. This may make it more difficult, but all the more important, to close the drinking water access gap between on- and off-reserve communities, especially given that Indigenous people in Canada make up the majority of the 1% of Canadians currently without access to improved drinking water [39] required for Canada to meet its Sustainable Development Goal target of universal access to drinking water (Target 6.1) [40].

### Study limitations

Some general methodological limitations were identified through this research process. First, the survey response rate (44/226; 19.5%) was lower than anticipated. Though surveys were disseminated to every adult, in an effort to give everyone a voice, many homes appeared to return one survey that was intended to be representative of the knowledge, actions, and beliefs of all adults in the residence. While response rates were incentivised through a draw for a tablet and potential respondents were reminded of the deadline, the desire to provide a household-level response was clearly stronger for some people. Additionally, as previously mentioned, discussions regarding a pipeline from the town were already under way during the data collection; this may have dis-incentivized people from participating as a potential solution had already been articulated. Secondly, while it was anticipated that some potential KIs may have felt uncomfortable participating or sharing sensitive stories because of the otherness of non-Indigenous members of the study team, some KIs may have been uncomfortable participating or sharing with the community research associate because this individual was also a community member. Allowing interviewees to choose which of the facilitators were or were not present during their interviews mitigated this limitation.

The low response rate as a sub-set of an already small population would suggest that the findings have limited generalizability within the community. However, during the co-analysis process with Chief and Council, many findings resonated with community challenges and experiences. Finally, although these findings represent the health, social, cultural, and economic costs and consequences to this particular First Nation, First Nations’ traditions and Traditional Knowledge varies. Thus, the results are not generalizable to all First Nations, but some elements are likely to resonate with other communities navigating life under a DWA.

## Conclusions

A key finding that emerged from this research is that DWAs negatively impact multiple facets of every-day life. These impacts vary according to age and gender and may affect an individual’s ability to consistently adhere to the advisory. This finding has implications for education and interventions. For example, women, and particularly mothers of young children, require tailored information to keep themselves and their families safe, as well as additional support to reduce the physical and time burdens placed upon them by DWAs. However, not all impacts were considered negative. Indeed, the existence of long-term DWAs drew attention from outside the community, which ultimately led to dialogues, resource allocation, and change. Some of this change was in response to this research project. Specifically, the findings were used to successfully advocate for improved drinking water for the community.

Additionally, benefits of participatory research and community ownership include enhanced local research capacity, and increased awareness of, and desire for, research to inform decisions. The elimination of DWAs on reserves remains a priority for the Government of Canada but needs to be expanded to include those who are not currently served by a community drinking water system. Communities will need to continue to advocate for their basic human rights to clean water and their rights as First Peoples to govern and guard their water and, by extension, the environment. The messaging that comes from research conducted in this way reminds leaders, funders, and partners that there are actual people with voices and stake in these issues – it is their health and culture on the line. There is power in the narrative that emerges because it humanizes decisions about local drinking water security.

## Supplementary Information

**Additional File 1. **Survey Instrument. This document provides a copy of the survey instrument distributed to eligible community members. The survey consisted of four short sections that focused on: 1. basic information about the respondent; 2. knowledge of the BWA; 3. water sources and uses before and during the BWA; and, 4. impacts of the BWA upon the respondent’s life.

**Additional File 2.** Interview Instrument. This document provides a copy of the interview instrument used to elicit contextual information from key informants and Elders. The interview instrument contains twelve interview questions or prompts.

## Data Availability

The datasets generated and/or analysed during the current study are owned and controlled by the First Nation community and are not publicly available. Requests to the community for access to these datasets may be initiated by contacting the corresponding author.

## Abbreviations

BWA: Boil water advisory
CBPR: Community-based participatory research
DWA: Drinking water advisory
HC: Health Canada
IDWPO: Indigenous drinking water projects office
ISC: Indigenous services Canada
KI: Key informant
TK: Traditional knowledge
