# Supplementary material for: “Is there anything good about a water advisory?”: an exploration of the consequences of drinking water advisories in an indigenous community
Source: BMC Public Health. 2020 Nov 13;20:1704. doi: 10.1186/s12889-020-09825-9 (PMC7666524; doi:10.1186/s12889-020-09825-9)
Supplement: Supplementary file 1 — Additional File 1. Survey Instrument. This document provides a copy of the survey instrument distributed to eligible community members. The survey consisted of four short sections that focused on: 1. basic information about the respondent; 2. knowledge of the BWA; 3. water sources and uses before and during the BWA; and, 4. impacts of the BWA upon the respondent’s life. [file 12889_2020_9825_MOESM1_ESM.pdf]

Additional File 1: Survey Instrument

Wauzhushk Onigum Nation Drinking Water Questionnaire (Community Members)

A: Personal Information

1. What is your gender? ☐ Male ☐ Female ☐ Prefer to not answer

2. How old are you?

☐ 18-29 yrs. ☐ 30-39 yrs. ☐ 40-49 yrs. ☐ 50 + yrs. ☐ Prefer to not answer

3. How many years has Wauzhushk Onigum Nation been your primary residence?

☐ < 1 yr. ☐ 1 to < 3 yrs. ☐ 3 to < 5 yrs. ☐ 5 to < 10 yrs. ☐ ≥ 10 yrs ☐ Prefer not to answer

4. Have you lived elsewhere for a substantial period of time (i.e., more than 6 months)?

☐ Yes ☐ No

4a. If yes, why?

____

____

5. What form of traditional education have you received?
Please describe:

____

OR ☐ None

6. In addition to your traditional education, what is the highest level of formal education that you have completed?

☐ Elementary school ☐ High school ☐ Vocational training
☐ University/college ☐ Graduate degree/professional credentials ☐ Prefer not to answer

B: Knowledge of Boiled Water Advisory (BWA)

7. How long have you lived under a BWA?

____

8. Based on your experience, please categorise the following statements as YES or NO during a BWA?

Applies only to my tap water	☐ YES	☐ NO	☐ I DON'T KNOW

I can drink my tap water as long as it is clear	☐ YES	☐ NO	☐ I DON'T KNOW
I should not drink my tap water	☐ YES	☐ NO	☐ I DON'T KNOW
I should boil my tap water to prepare my food	☐ YES	☐ NO	☐ I DON'T KNOW
I can use my tap water to make ice	☐ YES	☐ NO	☐ I DON'T KNOW
I can bathe / shower using my tap water	☐ YES	☐ NO	☐ I DON'T KNOW
I can give my babies and young children a sponge bath using my tap water	☐ YES	☐ NO	☐ I DON'T KNOW
Before preparing meals, it is okay to wash my hands with tap water	☐ YES	☐ NO	☐ I DON'T KNOW
I can use my tap water to clean my teeth	☐ YES	☐ NO	☐ I DON'T KNOW
I should boil my tap water before I drink it	☐ YES	☐ NO	☐ I DON'T KNOW
I should boil my tap water before I bath my babies and young children	☐ YES	☐ NO	☐ I DON'T KNOW
I can use a Brita filter to decontaminate my tap water	☐ YES	☐ NO	☐ I DON'T KNOW
I should drink bottled water	☐ YES	☐ NO	☐ I DON'T KNOW

9. How did you hear about the BWA in Wauzhushk Onigum Nation?

____

____

10. Is there anything else you would like to share concerning the BWA in Wauzhushk Onigum Nation?

____

____

____

____

C: Water Sources and Uses

11. Please identify (with a check mark) where you get your water for the following purposes when there isn't a BWA:

Purpose	Source				Not Applicable
	Tap	Lake	Bottled	Other (please describe)	
Drinking					

Food preparation					
Cooking					
Cleaning teeth					
Hand washing					
Bathing					
Cleaning					
Laundry					
Ceremonies					
Other use (please describe)					

12. Please identify (with a check mark) where you get your water for the following purposes under a BWA:

Purpose	Source				Not Applicable
	Tap	Lake	Bottled	Other (please describe)	
Drinking					
Food preparation					
Cooking					
Cleaning teeth					
Hand washing					
Bathing					
Cleaning					
Laundry					
Ceremonies					
Other use (please describe)					

D: Impact of BWA

13. Which of the following face(s) best describes how the BWA makes you feel? (Circle as many as apply.)

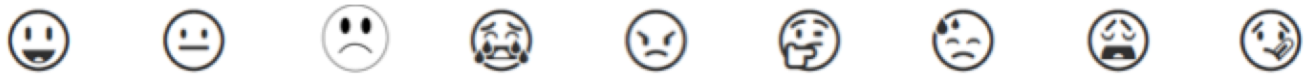

Happy Neutral Unhappy Sad Angry Pensive Worried Overwhelmed Sick

Other(s):

____

14. How seriously are you taking the current BWA (select one)?
    - ☐ Always adhere
    - ☐ Sometimes adhere
    - ☐ Adhere when convenient

☐ Rarely adhere
☐ Never adhere

15. How significant were any **negative** impacts that you experienced under a BWA?

	Significant Impact	High impact	Some impact	Little impact	No impact
Financially					
Physically (health)					
Psychologically					
Socially					
Spiritually					
Time Burden					

16. Please use this space if you would like to share additional information regarding these **negative** impacts:

___
___
___
___

17. Please describe any **positive** impacts of being under a BWA?

___
___
___
___

OR ☐ I cannot think of any **positive** impacts

18. Is there anything else you would like to say about water resources in your community and/or the BWA? Please use this space if you would like to share additional information:

___
___
___
