# Supplementary material for: “Is there anything good about a water advisory?”: an exploration of the consequences of drinking water advisories in an indigenous community
Source: BMC Public Health. 2020 Nov 13;20:1704. doi: 10.1186/s12889-020-09825-9 (PMC7666524; doi:10.1186/s12889-020-09825-9)
Supplement: Supplementary file 2 — Additional File 2. Interview Instrument. This document provides a copy of the interview instrument used to elicit contextual information from key informants and Elders. The interview instrument contains twelve interview questions or prompts. [file 12889_2020_9825_MOESM2_ESM.pdf]

Additional File 2: Interview Instrument

1. Can you tell us about your relationship to Wauzhushk Onigum Nation, particularly as it pertains to drinking water?

2. Have you been involved in the boil water advisories in Wauzhushk Onigum Nation in any way? If so, what was your role?

3. How do you think the boil water advisory affects the residents of Wauzhushk Onigum Nation?

4. Has there been any impact for you as an individual? If so, what?

5. Advisories can be very stressful and create tension in relationships. What has the period of the boil water advisory been like for you in terms of your relationship with people/others in Wauzhushk Onigum Nation?

6. What do you believe to be the critical problems resulting in the boil water advisories in Wauzhushk Onigum Nation?

7. Do you believe there is a solution to the water problems in Wauzhushk Onigum Nation?

8. What do you think is the best solution for the drinking water problems in Wauzhushk Onigum Nation?

9. What do you think it will take to achieve this outcome?

10. What do you think will be the actual outcome?

11. Is there any historic information about the relationship between Wauzhushk Onigum Nation and your organisation / department that would help us to better understand the current situation in Wauzhushk Onigum Nation?

12. Is there anything else that you think we should know about the drinking water situation and/or advisories in Wauzhushk Onigum Nation?
